# High Sensitivity of T-Ray for Thrombus Sensing

**DOI:** 10.1038/s41598-018-22060-y

**Published:** 2018-03-02

**Authors:** Chi-Kuang Sun, Hui-Yuan Chen, Tzu-Fang Tseng, Borwen You, Ming-Liang Wei, Ja-Yu Lu, Ya-Lei Chang, Wan-Ling Tseng, Tzung-Dau Wang

**Affiliations:** 1 Department of Electrical Engineering and Graduate Institute of Photonics and Optoelectronics, National Taiwan University, Taipei, 10617 Taiwan; 2 Molecular Imaging Center, National Taiwan University, Taipei, 10617 Taiwan; 3 Graduate Institute of Biomedical Electronics and Bioinformatics, National Taiwan University, Taipei, 10617 Taiwan; 4 Research Center for Applied Science and Institute of Physics, Academia Sinica, Taipei, 11529 Taiwan; 5 Department of Photonics, National Cheng-Kung University, Tainan, 70101 Taiwan; 6 Cardiovascular Center and Division of Cardiology, Department of Internal Medicine, National Taiwan University Hospital, Taipei, 10002 Taiwan

## Abstract

Atherosclerotic plaque rupture or erosion and subsequent development of platelet-containing thrombus formation is the fundamental cause of cardiovascular disease, which is the most common cause of death and disability worldwide. Here we show the high sensitivity of 200–270 GHz T-ray to distinguish thrombus formation at its early stage from uncoagulated blood. A clinical observational study was conducted to longitudinally monitor the T-ray absorption constant of *ex-vivo* human blood during the thrombus formation from 29 subjects. Compared with the control group (28 subjects) with uncoagulated blood samples, our analysis indicates the high sensitivity of 200–270 GHz T-Ray to detect thrombus with a low p-value < 10^−5^. Further analysis supports the significant role of platelet-activated thrombotic cascade, which modified the solvation dynamics of blood and occurred during the early coagulation stage, on the measured T-Ray absorption change. The ability to sense the thrombus formation at its early stage would hold promise for timely identification of patients at risk of various atherothrombotic disorders and save billions of lives.

## Introduction

Cardiovascular disease is the most common cause of death and disability worldwide and represents 30% of global deaths reported by Word Health Organization in 2010^1^. Atherosclerotic plaque rupture or erosion and subsequent development of platelet-containing thrombus formation is the fundamental cause of cardiovascular diseases including myocardial infarction, ischemic stroke, and embolic stroke^2^. Individual’s thrombogenic potential is determined not only by local factors like plaque morphology and stability, but also by systemic hemostatic factors comprising coagulation factors, fibrinolytic factors, and platelets^3^. Among these systemic factors, platelets are regarded to play a major role in driving atherothrombosis by localizing to lesions, delivering prothrombotic and proinflammatory molecules, and facilitating inflammatory cell infiltration into lesions^4^. Although different measures of various systemic factors have been shown to be predictive of cardiovascular events^5^, an *in situ* real-time monitoring system to be able to detect the local thrombus formation, especially at its early stage, as well as the traveling thrombus is critical for early warning of heart attack and stroke events. It is also of clinical importance to perform an integrative assessment of individual’s thrombotic potential given the multifactorial nature of atherothrombosis. Moreover, the temporal sequence of the hemostatic/thrombotic cascade remains largely unclear and awaits the development of sensitive means for early detection.

T-Ray, the terahertz (THz) electromagnetic waves^6^, is known to be sensitive to collective vibrational motions of biomolecules^7–12^. THz spectroscopy studies of human and animal blood had indicated the high sensitivity of THz waves to a few critical blood ingredients including triglycerides, red blood cells, and glucose^13–18^. This leads to the observation of a great difference, up to 15%^18^, in THz absorption properties of human blood. THz waves are also known to be sensitive to the dynamical reorientations of liquid water molecules’ dipole moments. When liquid water is surface-absorbed^19^, nano-confined^20^, or freezes into the solid phase as ice^21^, THz absorption decreases abruptly due to restricted orientation motion. Coagulation is the process of thrombus formation which turns blood from a liquid phase to a jelly-like phase through an avalanche of biochemical events initiated by platelets. Those cascaded biochemical events would largely affect the THz absorption and make THz wave a perfect candidate for the long-desired thrombus sensing device. We thus hypothesize THz waves as a solution to noninvasively sense the thrombus formation without labelling and as a potential means to assess individual’s thrombotic potential and to *in situ* monitor the hemostatic/thrombotic cascade.

In this work, we report that low-frequency sub-THz waves are with a high sensitivity to thrombus formation. Time-dependent THz absorption spectra of coagulating human blood from 29 subjects were measured *ex vivo* and were compared to the THz absorption spectra of uncoagulated human blood from another 28 subjects by using a portable THz spectrometer next to the cardiac catheterization suite, to ensure timely human blood coagulation observation right after blood extraction. High sensitivity to thrombus formation can be found in the sub-THz frequency range between 0.2–0.27 THz (n = 57, p-value < 10^−5^). This high sensitivity of low-THz waves is attributed to the modified water absorption due to platelet-activated thrombotic cascade during the early coagulation process. Combined with the recently demonstrated *in vivo* THz vessel imaging system^22^, also operated in the low-THz range around 300 GHz, our work opens the door for *in situ* noninvasive monitoring of not only the local thrombus formation at its early stage, to assess individual’s systemic hemostatic factors, but also the traveling thrombus in blood stream, for early detection of atherosclerotic plaque rupture and embolic stroke.

## Results

**Figure 1:**
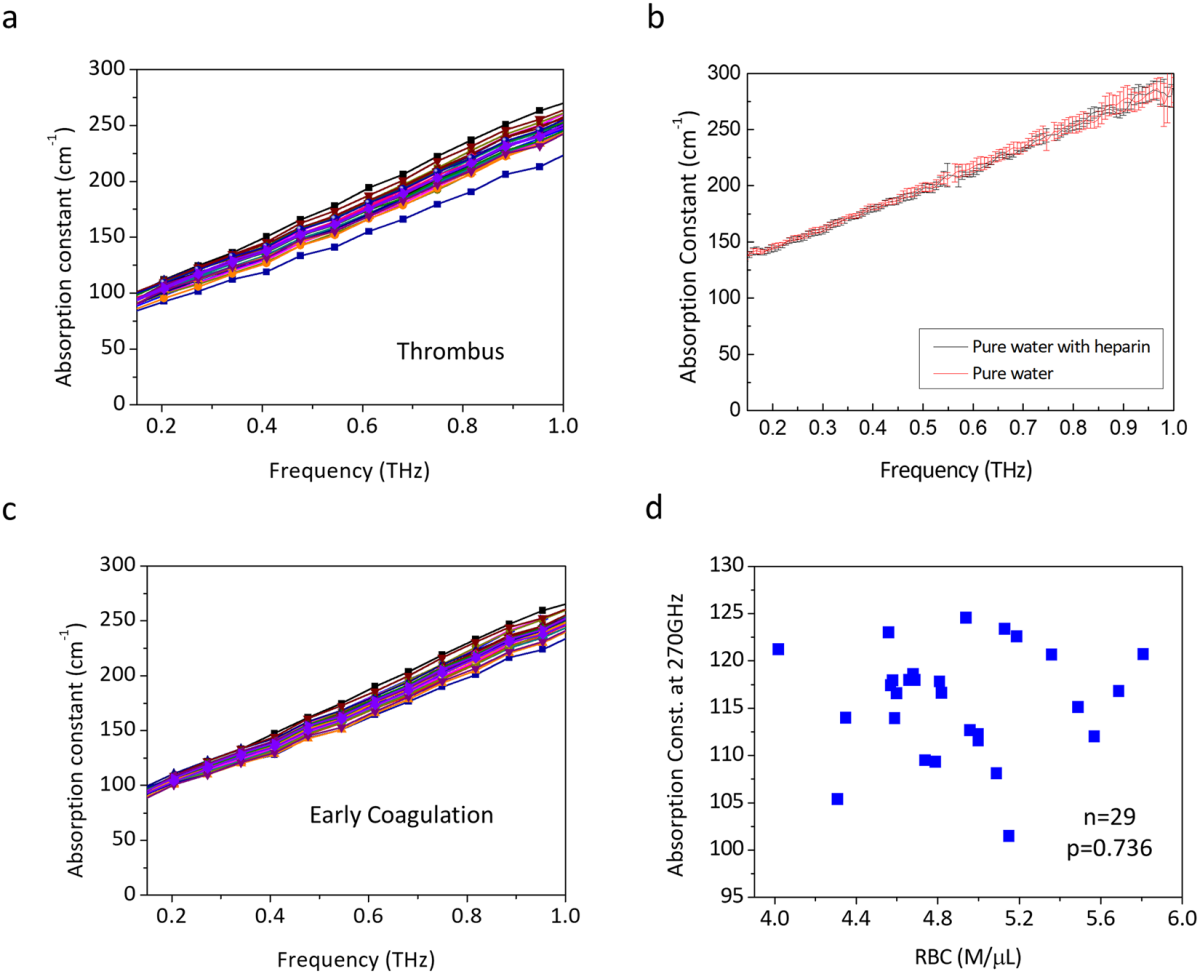
THz absorption spectra of human blood and water. (**a**) One-minute-averaged THz absorption spectra of human blood thrombus taken from 29 subjects. The spectra were taken at the first minute right after the blood samples had turned into thrombus. (**b**) THz absorption spectra of pure water (red line) and heparin-added water (black line). Data was summarized from 10 measurements. (**c**) One-minute-averaged THz absorption spectra of early coagulated human blood taken from 29 subjects. The spectra were taken at the first minute of the measurement, which started around 3 minute after the blood extraction. (**d**) One-minute averaged 270 GHz absorption constants measured right after the thrombus formation versus RBC count. No correlation was observed (n = 29, p-value = 0.736).

**Figure 2:**
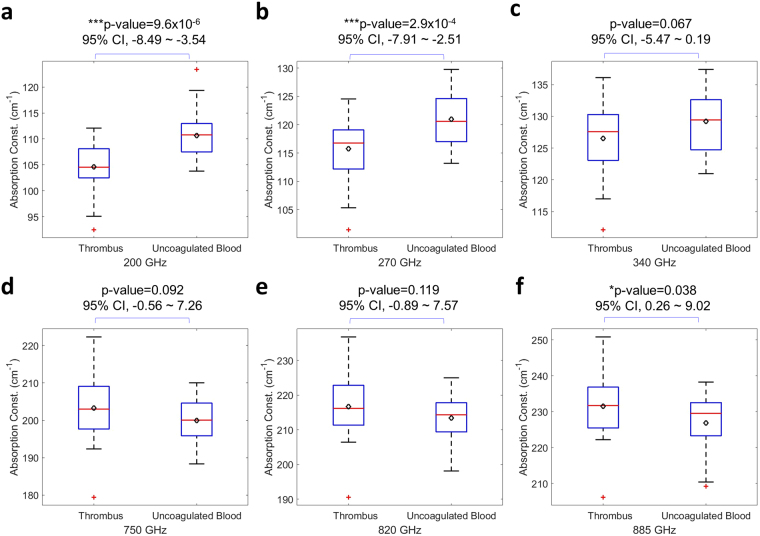
Statistical analysis results comparing the measured THz absorption constants of human thrombus from uncoagulated human blood. Two tailed unpaired T-test was performed between the one-minute-averaged THz absorption constant of thrombus shown in Fig. 1a (n = 29) and the one-minute averaged THz absorption constant of uncoagulated human blood (n = 28). Results on (**a**) 200 GHz, (**b**) 270 GHz, (**c**) 340 GHz, (**d**) 750 GHz, (**e**) 820 GHz, and (**f**) 885 GHz are shown, where the central red line indicates the median, the bottom and top edges of the box indicate the upper and lower quartiles, the whiskers extend to the most extreme data points not considered outliers, the red cross (+) symbol indicate the outliner, and the black diamond indicates the statistical mean values. P-values and confidence intervals (95% CI) for the difference in two groups are also denoted. *0.01 < p < 0.05; **0.001 < p < 0.01; ***p < 0.001.

We had performed continuous THz spectral measurement on coagulated blood from 29 patients (see Methods). The THz spectra were continuously acquired every 0.5 second for 30 minutes. Blood clotting time was found to range between 8 and 21 minutes, including the 3-minute-interval between the blood extraction and the beginning of the THz spectrum measurement. The absorption constants of various sub-THz frequencies acquired at the 1^st^ minute right after the blood turned into thrombus, shown as Fig. 1a, were compared to a control group where heparin was added into the extracted human blood to prevent coagulation. The control group data, which was the one-minute average spectra with another 28 different subjects, were taken from a previous study^18^ performed under exactly the same experimental setting and conditions (see Supplementary Information), while the data were blended into this current experimental data and re-analyzed not just for blinding purpose but also to avoid any possible system error. An unpaired T-test, as summarized in Fig. 2, indicates the high sensitivity of 200–270 GHz THz waves to distinguish thrombus, or the so-called blood clot, from uncoagulated blood (n = 57; p < 3 × 10^−4^). A greater than 5 cm^−1^ reduction on the THz absorption constant can be observed between thrombus and the uncoagulated blood. For T-test results across the whole measured spectrum range (130–1020 GHz), please refer to Supplementary information, and some of the results were presented also in Fig. 2, especially the results around 820 GHz.

Further studies were conducted on the possible origin regarding the observed high sensitivity of the 200–270 GHz THz waves to distinguish thrombus from uncoagulated blood. We first examined the possible influence of heparin, which was added to the control group to prevent blood coagulation. THz time domain spectroscopy measurement^23^ on pure water with and without heparin with a concentration of 15 USP unit/ml, same as the control experiment, was performed to investigate the influence of heparin to the measured THz spectra. Measurement was repeated for 10 times with the same microfluidic device designed for blood spectrum acquisition and the results indicated that the observed absorption constant change due to the thrombus formation cannot be attributed to the influence of the heparin itself to the measured THz waves. As shown in Fig. 1b, our repeated experiments indicated that the influence of the 15 USP unit/ml heparin itself to THz absorption is well-within our measurement error throughout the whole THz spectral regime under study.

**Figure 3:**
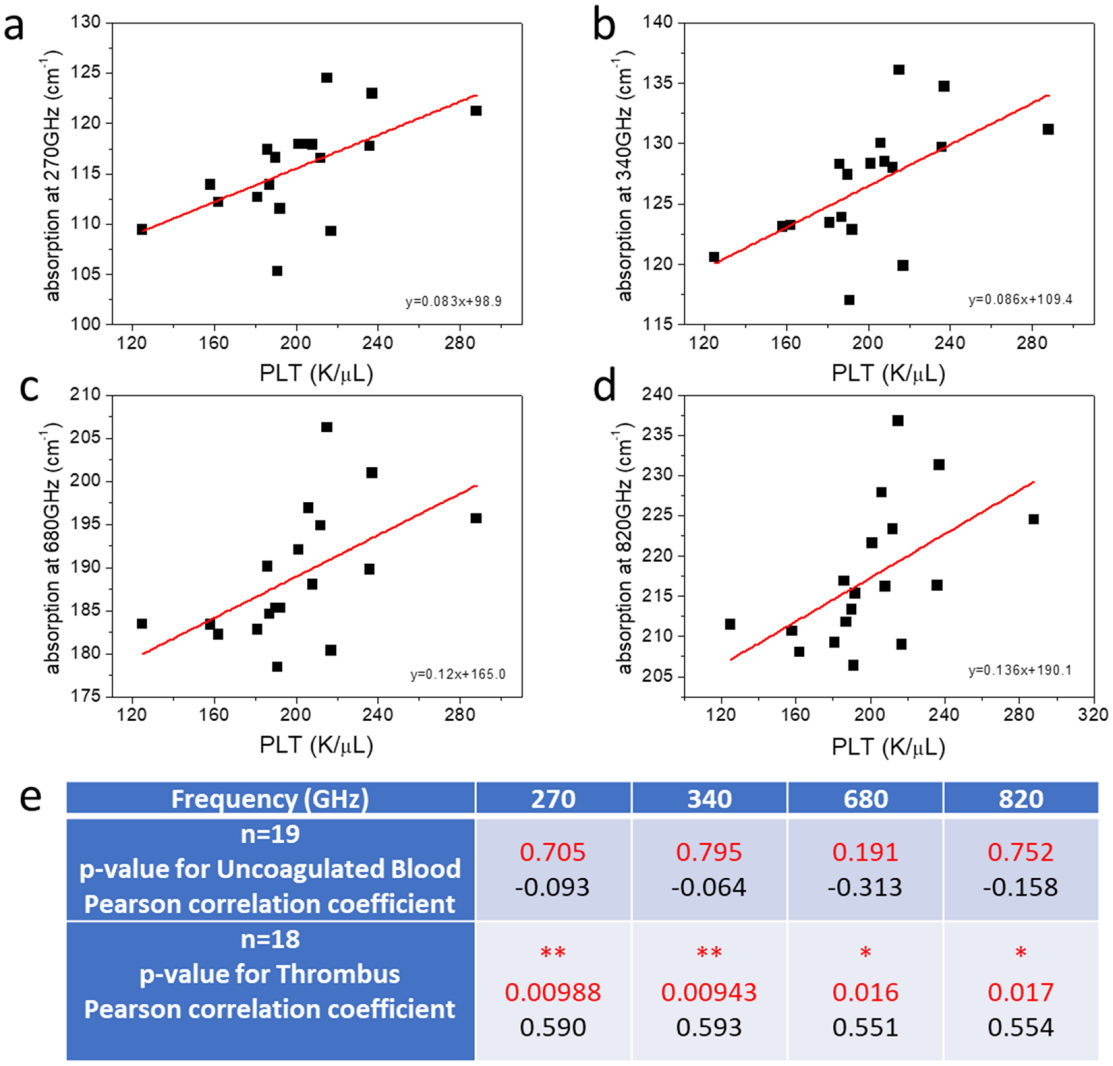
THz absorption constant of thrombus correlates with PLT concentration. One-minute averaged THz absorption constants (n = 18) measured right after the thrombus formation versus PLT concentration for (**a**) 270 GHz, (**b**) 340 GHz, (**c**) 680 GHz, (**d**) 820 GHz. Significant correlation was observed for all analyzed frequencies. (**e**) Statistical analysis results summarizing the p-value and the Pearson coefficient for the correlation studies between THz absorption and uncoagulated human blood (n = 19) or human blood thrombus (n = 18). *0.01 < p < 0.05; **0.001 < p < 0.01; ***p < 0.001.

Our previous investigation on the uncoagulated human blood^18^ has indicated that red blood cell (RBC) count dominantly affected the 270 GHz THz absorption constant. Since RBCs are involved in blood clotting and play an active role in normal and pathologic hemostasis^24^, we thus performed the bivariate Pearson correlation analysis (see Methods) between the RBC count and the absorption constant at 270 GHz, as shown in Fig. 1d. The one-minute averaged 270 GHz absorption constants measured right after the thrombus formation were not correlated with the RBC count (n = 29, p-value = 0.736), distinct from the previous result with a p-value of 0.044 (n = 28) for uncoagulated blood^18^. Our study thus indicates a different THz nature of blood after the initiation of the coagulation event so that RBC count no longer dominates the low-THz wave absorption. Among different systemic hemostatic factors, platelets are regarded to play a major role in driving atherothrombosis by localizing to lesions, delivering prothrombotic and proinflammatory molecules, and facilitating inflammatory cell infiltration into lesions^5^. We thus analyzed the correlation between the one-minute averaged THz absorption constant and the corresponding platelet (PLT) concentration, as shown in Fig. 3. In this investigation, RBC count was limited within a narrower region in 4 < RBC count < = 5 M/μL to avoid significant absorption offset caused by the wide variety of RBC volume fraction, which could be up to 45%. PLT count was limited within the normal region^25^ of 100 K/μL to 300 K/μL to avoid the influence of patients with abnormal coagulation function. 19 and 18 samples were respectively selected from uncoagulated blood (control group) and thrombus groups with these two limitations. Due to the low volume ratio of platelets in blood, our analysis on the uncoagulated human blood has indicated no correlation of platelet count to the measured THz absorption constant throughout the whole sub-THz range (n = 19, p = 0.705, Fig. 3e). However, as Fig. 3 shows, the 270 GHz absorption constant of the thrombus-forming blood had a positive correlation to the platelet count (n = 18, p-value = 0.00988, r = 0.59). This positive correlation exists across the whole investigated THz frequencies, as shown in Fig. 3, with a stronger correlation at the lower frequencies (for examples 270 vs. 820 GHz). For details, please refer to Supplementary Information.

Platelet is a type of blood cells occupying only a negligible fraction of volume in blood. Blood coagulation begins instantly after damaging the vessel endothelium, which in turn initiates the changes of the platelets^26–28^. During the early stage of coagulation process, the activated platelets will release the contents of stored granules into the blood plasma and activate more platelets^27^. These granules will also activate a G_q_-linked protein receptor cascade, activates protein kinase C, and phospholipase A_2_^28^. Activated platelets form a platelet plug, which further activates coagulation factor protein^25^. In the report of G.V.R. Born *et al*.^29^, the platelet activation time is on the order of 0.1 second. From the report of L. Zhou *et al*.^30^, more than 95% platelets aggregate in *ex-vivo* blood within 3 minutes. N.G. Ardlie *et al*. also indicated^31^ that most platelet aggregation responses complete within 3 minutes at both the body temperature (37 °C) and at 20 °C. The later clotting process that turns the whole blood to the jelly-like thrombus took much more time.

**Figure 4:**
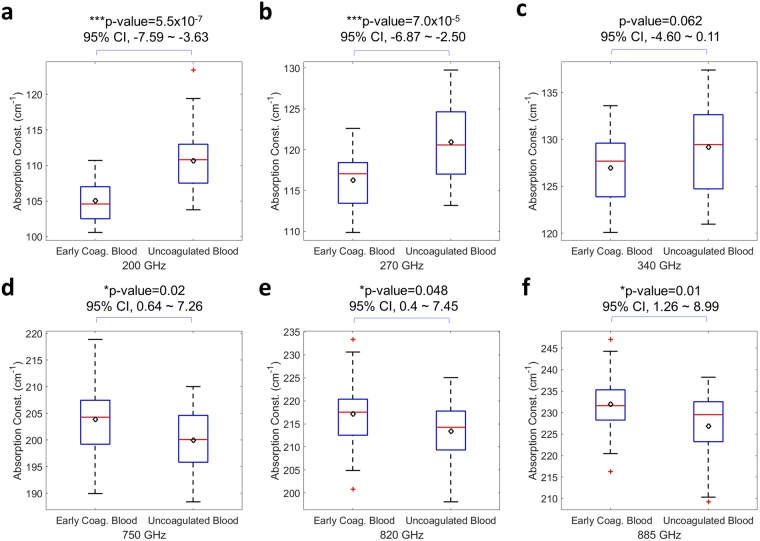
Statistical analysis results comparing the measured THz absorption constants of early coagulated human blood from uncoagulated human blood. Two-tailed unpaired T-test was performed between the one-minute-averaged THz absorption constant of early coagulated human blood shown in Fig. 1c (n = 29) and the one-minute averaged THz absorption constant of uncoagulated human blood (n = 28). Results on (**a**) 200 GHz, (**b**) 270 GHz, (**c**) 340 GHz, (**d**) 750 GHz, (**e**) 820 GHz, and (**f**) 885 GHz are shown, where the central red line indicates the median, the bottom and top edges of the box indicate the upper and lower quartiles, the whiskers extend to the most extreme data points not considered outliers, the red cross (+) symbol indicate the outliner, and the black diamond indicates the statistical mean values. P-values and confidence intervals (95% CI) for the difference in two groups are also denoted. *0.01 < p < 0.05; **0.001 < p < 0.01; ***p < 0.001.

To further test if thrombus formation changed THz absorption through the early stage platelet activation and amplification process, we analyzed the one-minute-averaged THz absorption constant measured starting at 3 minutes after the extract of blood (Fig. 1c), when the early stage platelet amplification process completed while blood had not formed the jelly-like thrombus. The unpaired T-test to the uncoagulated blood group, as summarized in Fig. 4, indicates the high sensitivity of 200–270 GHz THz waves to distinguish early-coagulated blood (n = 29) from uncoagulated blood (n = 28). Compared with the results shown in Fig. 2, significant p-values can be observed not only in the 200–270 GHz range, but also in the 750–885 GHz range, despite of an opposite direction of changes in absorption constants. This finding supports the dominant role of the early stage platelet activation and amplification process to the modification of the THz absorption constant observed in thrombus. For T-test results across the whole measured spectrum range (130–1020 GHz), please refer to Supplementary Information.

## Discussion

Our clinical finding has a profound meaning. With a research boom in various T-Ray technologies, a killer application, which has not been found, is definitely needed for the THz-biomedicine field. Noninvasive remote sensing technology for early detection of thrombus formation, to be part of the future wearable devices, is urgently needed since atherosclerotic plaque rupture or erosion and subsequent development of platelet-containing thrombus formation is the fundamental cause of cardiovascular disease. The ability to sense the thrombus formation at its early stage would enable us to timely identify patients at risk of evolving atherothrombotic diseases and save billions of lives. A recent *in vivo* near-field T-Ray imaging studies on mice^22^ has demonstrated the capability of low-frequency T-Ray, operating at 340 GHz, to penetrate through the animal tissues and to longitudinally and quantitatively monitor *in vivo* the THz absorption constant of blood noninvasively. With the recent advance on miniaturized sub-millimeter wave source and detectors^13^, the 200–270 GHz based early thrombus warning device will be a solution to the number one killer in the world and will definitely attract attentions across the medical engineering field.

It is also of clinical importance to develop a methodology for integrative assessment of individual’s thrombotic potential given the multifactorial nature of atherothrombosis. A few optical^32,33^ and ultrasound^34^ imaging methods have been applied to investigate blood coagulation. Optical methods are useful to observe the plug-forming process in plasma based on the scattering measurement. Ultrasound methods are useful to measure the coagulating blood viscosity and the formed plug size^34^. All these methods are with a sensitivity to the final plug forming process but not the early thrombotic cascade, distinct from THz waves. As more detailed investigations will be needed, our study suggests a high sensitivity of low-frequency THz waves to the outcome of the platelet activation and amplification-cascade process, thus also indicates the high potential of THz waves as a means to assess individual’s thrombotic potential, in which platelets play a pivotal role.

A recent concentration-dependent study on protein water solution^35,36^ with proteins of different molecular weights by using the WR-3 waveguide band (220–350 GHz) has indicated the primary role of the hydration layers on the reduction of the THz absorption with increased protein concentration. Compared with similar studies by using higher frequency THz waves^37–40^, this recent study indicated a higher sensitivity of 220–350 GHz waves for the influence of proteins on the properties of the surrounding water. This agrees with the trend that we observed in Fig. 3, which shows a stronger correlation of the measured THz absorption with platelets in the 270–340 GHz than those in the higher frequencies. With a much stronger sensitivity to the platelet-activation resulted protein-generation cascade in blood plasma, 200–270 GHz waves are more capable to overcome the individual’s variation which would also influence the measured THz absorption constant in blood.

**Figure 5:**
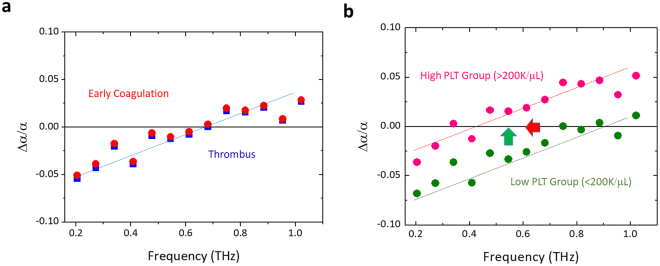
Solvation dynamics affect terahertz absorption after coagulation. (**a**) Normalized absorption change spectra Δα/α_blood_ for early coagulated blood (red solid circles) and thrombus (blue solid squares), where Δα is defined as the difference between the average absorption constant of early coagulated blood α_early_ (n = 29) and that of uncoagulated blood α_blood_ (n = 28). After the platelet-activation resulted thrombotic cascade, THz “excess” & “defect” behaviors were observed for the frequency above & below a transparency frequency (680 GHz). Blue dotted line is a guide to our eye for this behavior. (**b**) Normalized absorption change spectra Δα/α_blood_ for early coagulated blood with high-PLT-count (200 K/μL-300 K/μL; n = 9, red solid circles) and low-PLT-count (100 K/μL-200 K/μL; n = 9, green solid circles) groups. RBC count was limited within a narrow region (4 < RBC count < = 5 M/μL). Red and green dotted lines are guides to our eye to illustrate different transparency frequencies for these two PLT groups. Higher PLT concentration resulted in red shift (red arrow) of the transparency frequency and increased (green arrow) THz absorption.

Besides the 200–270 GHz responses, we have also noticed the reduction on the measured THz absorption constant for the frequency range of 200–400 GHz and the increase on the measured THz absorption constant for the frequency range of 750–1020 GHz, when comparing the early-coagulated blood to uncoagulated blood (Supplementary Information). Figure 5a shows the normalized absorption change for early coagulated blood Δα/α_blood_, where Δα is defined as the difference between the absorption constant of early-coagulated blood α_early_ and that of uncoagulated blood α_blood_. As a result, after the platelet-activation resulted thrombotic cascade, a THz “excess” behavior^38–40^ was observed for the frequency above the transparency frequency which is 680 GHz and a THz “defect”^38–40^ behavior was observed for the frequency below this transparency frequency. Here the transparency frequency is defined as the frequency where Δα equals zero; THz excess is defined as increased absorption compared to uncoagulated blood, while THz defect is defined as decreased absorption compared to uncoagulated blood. Even though blood plasma contains dissolved proteins, glucose, and electrolytes, blood plasma is mostly composed by water with a volume fraction up to 95%. Water and its unique role in life has been listed as one of the top challenges in chemistry by G. M. Whitesides^41^. Recent studies have indicated the active roles of water molecules in many processes that are essential to life^42^ besides just a passive solvent. For macromolecules like proteins, the hydration shell that covers the surface is critical to the stability of its structure and function. In the THz frequency range, the collective femtosecond and picosecond hydrogen bond dynamics of water and the large amplitude motions of biomolecules come into play^38^. THz waves are thus highly sensitive to protein-solvation dynamics, as indicated by many previous researches^35–40^. With a thrombotic cascade, rich protein-solvation dynamics occur during the early coagulation period, and THz waves should thus be sensitive also to this complicated thrombotic cascade process, as proven by our experimental summation. Recent studies on solvated mono- and disaccharides by Havenith and her co-workers had shown the strong coupling between solute and solvent modes, which results in an increase of THz absorption for frequency above 1.5 THz and a decrease of THz absorption for frequency below 1.5 THz^38–40^. This was attributed to the frequency shift of the vibrational density of states of the solvent modes through a numerical simulation. Even though such a quantitative numerical analysis is currently unavailable for blood coagulation analysis due to the high degree of complexity and due to too many unknown parameters, we suggest that our observed THz excess and THz defect behavior around 680 GHz could be attributed to the modified coupling between different solutes (proteins) and solvent (water) modes due to the platelet-activated thrombotic cascade process, which involves multiple plasma proteins (coagulation factors), PLTs, and RBCs that work together through a cascade of biochemical processes. It is important to notice that this is for the first time the protein solvation dynamics was observed under a real physiological condition. Our further analysis, as shown in Fig. 5b, also indicates a solute concentration dependency on the observed transparency frequency, which in average was around 680 GHz for early-coagulated blood. Similar to previous analysis here RBC count was limited within a narrow region (4 < RBC count < = 5 M/μL) while the normalized absorption spectra for early coagulated blood Δα/α_blood_ was plotted for two PLT count groups, 100 K/μL–200 K/μL (n = 9; average 175 K/μL; green solid circles) & 200 K/μL–300 K/μL (n = 9; average 224 K/μL; red solid circles). For both PLT groups, a linear increase of Δα/α_blood_ versus THz frequency can be observed, as indicated by the dotted lines as the guide to our eyes. On the other hand, a significant red shift of the transparency frequency from 0.9 THz for the lower PLT count group to 0.4 THz for the higher PLT count group can be observed. This significant red shift (red arrow in Fig. 5b) of the transparency frequency versus increased PLT count, −0.5 THz for 50 K/μL, is responsible for the observed positive correlation (green arrow in Fig. 5b) between THz absorption of thrombus and PLT count across the whole measured spectral range (130–1020 GHz, see Supplementary Information, Table E), as illustrated and indicated by Fig. 3. As also summarized in Fig. 5a, the later clotting process does not make significant shift on the transparency frequency, with a minor blue shift which in turn causes a mild reduction on the measured absorption constant. It is important to notice that there is no absorption peak observed either in our measured absorption spectra (shown in Fig. 1a and c), or in our normalized absorption spectra Δα/α_blood_ (shown in Fig. 5). We have observed some weak fluctuation of the experimental data point in Fig. 5. This could be attributed to the residue Fabry-Perot etalon effect even after the strong absorption attenuation (on the order of e^−2^ to e^−4^) in blood in our 100 μm-thick fluidic-sample chamber, which could results in a ~600 GHz free spectral range. This weak etalon effect appears at the same frequencies for two PLT groups as shown in Fig. 5a and b, and is not responsible for the observed red shift of the transparency point. This weak effect does not concern our analysis conclusion, either in our frequency dependent studies like Fig. 5 or in our comparison analysis between coagulation and uncoagulation blood, since they both experience the same effect by using the same fluidic chambers while the weak effect was much smaller than the fluctuation between individual subjects.

## Conclusion

Atherosclerotic plaque rupture or erosion and subsequent development of platelet-containing thrombus formation is the fundamental cause of cardiovascular disease, which is the most common cause of death and disability worldwide. In this study, we show the capability of 200–270 GHz T-Ray to distinguish thrombus formation at its early stage from uncoagulated blood. A clinical observational study was conducted to longitudinally monitor the T-Ray absorption constant of *ex-vivo* human blood during the thrombus formation from 29 subjects. Compared with another control group of uncoagulated blood samples with 28 subjects, our analysis indicates the ultra-high sensitivity of 200–270 GHz THz waves to detect thrombus with a low p-value < 10^−5^. Further analysis supports the significant role of platelet-activated thrombotic cascade, which occurred during the early coagulation stage, on the measured THz absorption change. We attribute the observed high T-Ray sensitivity to the solvation dynamics during the platelet-activated thrombotic cascade, which results in frequency shift of the vibrational density of states of the water solvent modes. It is not only for the first time such a THz protein solvation dynamics was observed under a real physiological condition, the demonstrated ability to sense the thrombus formation at its early stage would also hold promise for timely identification of patients at risk of various atherothrombotic disorders and save billions of lives.

## Methods

### THz TDS System Setup

A portable THz time-domain spectrometer (mini-Z, Zomega Inc.) was placed in the laboratory right next to the cardiac catheterization suite of National Taiwan University Hospital, to ensure fresh human blood spectrum acquisition immediately after blood extraction from every experimental subject. The portable THz time-domain spectrometer used an external fiber-coupled 1.5-μm pulsed laser with a 140-mW average power, with less than 100-fs pulse duration, and with a pulse repetition rate of 100 MHz as the pump source that was split into a pump and a probe beam for THz wave generation and detection. A large-aperture photoconductive antenna was used to generate the broadband THz wave and an electro-optic crystal was used to detect the THz waveform. The system measured the THz spectrum from 0.1 THz to 3 THz. Each waveform was acquired in 0.5 second, and the peak dynamic range was greater than 48 dB under the 0.5 second acquisition condition. To improve the signal-to-noise ratio (SNR), we presented the 1-minute waveform by averaging 120 continuous 0.5-second waveforms in sequence numerically. The fluidic-sample chamber^17^ for transmission-type measurement was self-designed with 2 polyethylene (PE) windows, one was a flat PE cap and the other was with a 100 μm-thick 1.5 cm-wide fluidic channel at one side. The PE window and the PE cap were sandwiched by two aluminum frames and locked by four nuts, and the injected liquid can thus be sealed. All the experiments were operated at a room temperature of 24 °C with a stable humidity 60%. In each time of the measurement, the reference spectrum of the empty chamber was acquired one minute before the sample injection. The measurement repeatability was tested by injecting bulk water, with the chamber assembled, fixed, water injected, and disassembled three times. The ratio of the standard deviation (STD) to the average (AVG) was less than 1% in all frequencies between 0.1–1.2 THz. The system fluctuation was also proved to be negligible when compared with the measured difference between samples.

### Clinical protocol

This study was conducted according to the Declaration of Helsinki Principles, and the following protocol was approved by the Institutional Review Board of National Taiwan University Hospital (clinical trial registration number: 200912038 R). Informed consent was obtained from each subject prior to study entry. The average age of 29 patients was 65 ± 10 years old, and 2 of them were female. All of them had stable coronary artery disease and had been treated with both aspirin and clopidogrel. None of them had been treated with any oral anticoagulants. No restriction on gender, age, race, or blood-related factor were applied in the recruitment of clinical patients. The human blood samples were obtained before cardiac catheterization, and all patients followed 8-hour fasting guidelines before the procedure. 29 blood samples without adding any anticoagulant were collected and then injected into empty Eppendorf tubes. We started to acquire the reference spectra from the empty chamber one minute before the blood injection from the filled Eppendorf tube. The remaining blood inside the Eppendorf tube was used to check the clotting process so as to record the clotting time. We started THz spectrum acquisition around 3 minutes after blood extraction from patients. Spectra of each blood sample without anticoagulant was continuously recorded for 30 minutes, even longer than the time interval to form blood clot. Water evaporation during experiment is not allowed in our fluidic chamber, which matched the blood clotting situation inside the living objects. The volume fraction of the blood cells and plasma as well as the water content thus did not vary because of the sealed fluidic chamber. We recorded the time that the blood sample changed to the jelly-like phase by slightly shaking the Eppendorf constantly. Blood clotting time ranged between 8 and 21 minutes, including the 3-minute interval between the blood extraction and the beginning of the THz spectrum measurement. We also checked the state of the blood in the THz measurement chamber after the 30-minute measurements, and confirmed that all blood in chamber became solid as that left in Eppendorf. We thus assumed that during coagulation the time that blood in Eppendorf turned into thrombus was the same as the blood inside the THz measurement chamber.

The hematological variables, including red blood cell (RBC) count (M/μL), white blood cell (WBC) count (K/μL), mean corpuscular hemoglobin (MCH) (pg), mean corpuscular hemoglobin concentration (MCHC) (g/dL), and platelet (PLT) count (K/μL) were examined one day before the cardiac catheterization.

### Analysis protocol

To analyze the measured THz dielectric properties of blood, simple calculations were applied and are described below^17^. The spectrum of the THz electric field transmitted through the empty container *E*_*ref*_(ω) was:

1 $${E}_{ref}(\omega )={E}_{s}(\omega )\cdot {\rm{T}}{(\omega )}_{(PE-air)}\cdot {e}^{i\omega d/c}\cdot {\rm{T}}{(\omega )}_{(PE-air)},$$

where *E*_*s*_(ω) is the electric field of the THz source, ω is the THz angular frequency, *d* is the thickness of the fluidic channel, and *c* is light speed; whereas the electric field transmitted through the container with blood *E*_*sample*_(ω) was:

2 $${E}_{sample}(\omega )={E}_{s}(\omega )\cdot {\rm{T}}{(\omega )}_{(PE-blood)}\cdot {e}^{i\omega d\cdot n(\omega )/c}\cdot {e}^{(-\omega d\cdot \kappa (\omega )/c}\cdot {\rm{T}}{(\omega )}_{(blood-PE)},$$

where n(ω) and κ(ω) are the real and imaginary parts of the refractive index of blood. T(ω) _a–b_, which is the transmission coefficient from medium a to medium b, has the form of: $\,\frac{2\overline{{n}_{a}}}{\overline{{n}_{a}}+\overline{{n}_{b}}}$, where $\overline{{n}_{a}}={n}_{a}+i{\kappa }_{a}$ is the complex refractive index. The THz absorption in air was neglected. The refractive indices of PE^43^ and air were 1.5 and 1, respectively, and the absorption of PE was negligible. The complex refractive index of blood was first obtained approximately by^17^:

3 $${\kappa }_{blood}(\omega )\cong (\frac{\mathrm{ln}({|\frac{{E}_{sample}(\omega )}{{E}_{ref}(\omega )}|}^{2})}{liquid\,thickness\,(d)})\cdot c/2\omega ,$$

and

4 $${n}_{blood}(\omega )\cong ({\rm{phase}}\,{\rm{difference}}({\rm{\Delta }}{\varphi }_{sample-ref}))\cdot c/\omega d+1.$$

Then the approximate n_blood_ and κ_blood_ calculated by equation (3) were substituted back to equation (2). The transmission coefficients through interfaces can thus be eliminated to keep the exponential term and to calculate new n_blood_ and κ_blood_. By repeating this process several times, the n_blood_ and κ_blood_ will converge to fixed values. As the converged n_blood_ and κ_blood_ values had less than 0.001 variation, the iteration process stopped. The absorption coefficient α has the form of 2·*ω*·*κ*(*ω*)/*c*.

All original THz time domain traces with exactly the same format from the control group and from the coagulation experiments were randomized and de-identified before a blinded absorption coefficient calculation. The absorption coefficient α was calculated by an analyzer (HYC), who was blinded to the origins of individual THz traces, using the same customized code.

For the unpaired T-test between coagulated blood (n = 29) and uncoagulated blood (n = 28), two-tailed two-sample T-test was performed using MATLAB R2015b (Mathworks, Natick, MA) function “ttest2”, where the p-value is one if the test rejects the null hypothesis at the 5% significance level, and zero if otherwise. In the unpaired T-test analysis, the absorptions measured at different time points or settings were considered to have a difference if the two-tailed p-value was lower than 0.05 (*0.01 < p < 0.05; **0.001 < p < 0.01; ***p < 0.001). Please noted we also tested the normality of our data (see Supplementary Information). For bivariate Pearson correlation analysis between the examined hematological variables and the absorption coefficients, Pearson’s correlation coefficients and p-values for the hypothesis of no correlation against nonzero correlation were conducted using MATLAB R2015b (Mathworks, Natick, MA) function “corr”. The blood absorption was considered to have a correlation with the examined hematological variables only if the p-value was less than 0.05 (*0.01 < p < 0.05; **0.001 < p < 0.01; ***p < 0.001).

### Data availability statement

The THz spectra datasets generated and analyzed during the current study are available from the corresponding authors on reasonable request.

### Code availability

MATLAB R2015b was used to calculate the THz absorption constant and conduct statistical analysis. Custom codes are available here: https://github.com/sun-ntu/Codes-for-Scientific-Reports-2018.

## Electronic supplementary material

Supplementary Information
