# Supplementary material for: High Sensitivity of T-Ray for Thrombus Sensing
Source: Sci Rep. 2018 Mar 2;8:3948. doi: 10.1038/s41598-018-22060-y (PMC5834502; doi:10.1038/s41598-018-22060-y)
Supplement: Supplementary file 1 — Supplementary Information [file 41598_2018_22060_MOESM1_ESM.pdf]

Supplementary Information

High Sensitivity of T-Ray for Thrombus Sensing

C.-K. Sun,[1,2,3,4]* H.-Y. Chen,[1,2] T.-F. Tseng,[1,2] B. You, [1,2] M.-L. Wei,[1,2] J.-Y. Lu,[5] Y.-L. Chang,[5] W.-L. Tseng,[5] T.-D. Wang[2,6]*

1. *Department of Electrical Engineering and Graduate Institute of Photonics and Optoelectronics, National Taiwan University, Taipei 10617, Taiwan*
2. *Molecular Imaging Center, National Taiwan University, Taipei 10617, Taiwan*
3. *Graduate Institute of Biomedical Electronics and Bioinformatics, National Taiwan University, Taipei 10617, Taiwan*
4. *Research Center for Applied Science and Institute of Physics, Academia Sinica, Taipei 11529, Taiwan*
5. *Department of Photonics, National Cheng-Kung University, Tainan 70101, Taiwan*
6. *Cardiovascular Center and Division of Cardiology, Department of Internal Medicine, National Taiwan University Hospital, Taipei 10002, Taiwan*

**Corresponding author: sun@ntu.edu.tw; tdwang@ntu.edu.tw*

Acquisition of the control group data

The following details were disclosed in a previous publication [A1].

The control group study was conducted according to the Declaration of Helsinki Principles, and the following protocol was approved by the Institutional Review Board of National Taiwan University Hospital. Informed consent was obtained from each subject prior to study entry. The human blood samples were obtained before cardiac catheterization, and all patients followed 8-hour fasting guidelines before the surgery. We started THz spectrum acquisition 3-4 minutes after blood extraction from patients, which was the shortest duration we could reach. 28 extracted blood samples were injected into heparin vacutainer (BD vacutainer). Heparin, having a high negative charge density, is the most common anticoagulant added in blood for examinations. The heparin concentration added was 15 USP (US Pharmacopeia) units of heparin per milliliter of blood. In each measurement, after the reference spectrum of the empty chamber was acquired for one minute, the human whole blood was injected and its spectrum was recorded 2 spectra/second continuously also for 1 minute.

Normality test of absorption constants of thrombus, early-coagulated blood and uncoagulated blood

We conduct both graphic method and numerical method for the normality test.
<u>Graphic method:</u>
For each blood group, i.e. thrombus, early-coagulated blood and uncoagulated blood, we plotted the histograms of the absorption constants at each THz frequency using MATLAB R2015b (Mathworks, Natick, MA) function “histfit”, as shown in Fig. S1, Fig. S2, and Fig. S3, respectively.

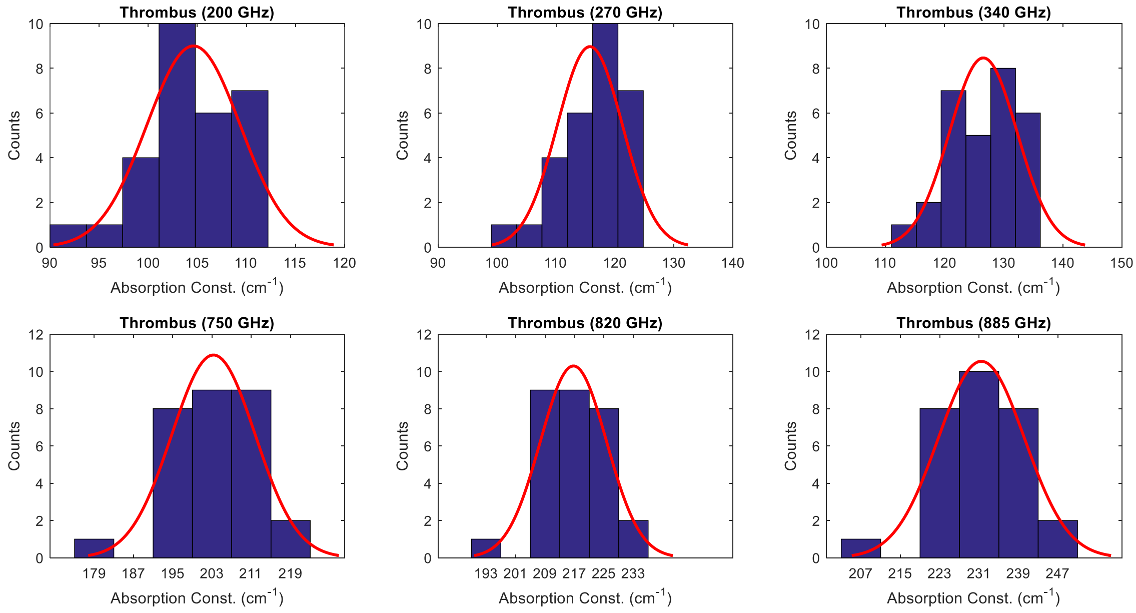

Figure S1. Thrombus group’s histograms of absorption constants at different THz frequencies.

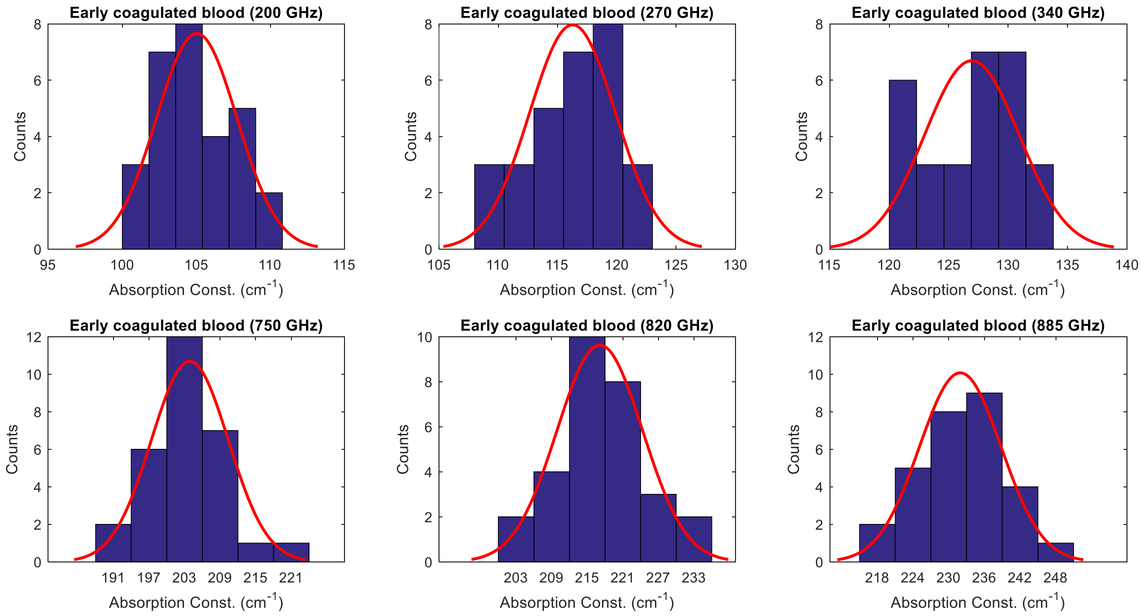

Figure S2. Early-coagulated blood group’s histograms of absorption constants at different THz frequencies.

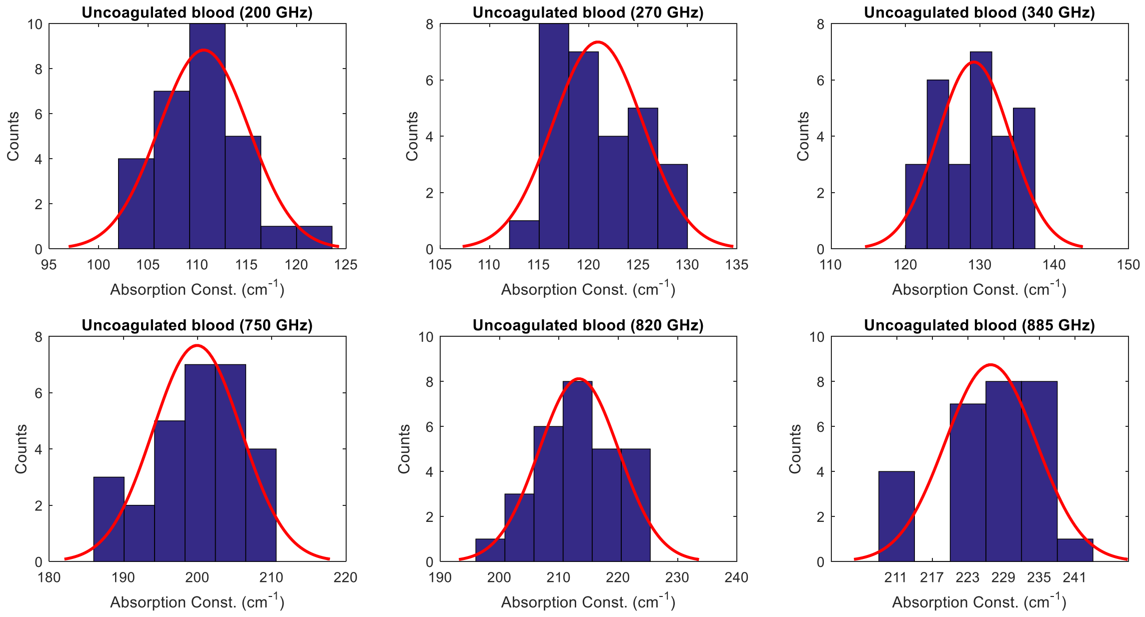

Figure S3. Uncoagulated blood group's histograms of absorption constants at different THz frequencies.

<u>Numerical Method:</u>

We conduct numerical normality test using Origin 8.0 (OriginLab, Northampton, MA) function. (Statistics -> Descriptive Statistics -> Normal Test)

The results of Kolmogorov-Smirnov (K-S) tests showed, at the 0.05 level, the data for all frequencies in all blood groups were significantly drawn from a normally distributed population.

T-test results across the whole measured spectrum range (130-1020GHz) comparing thrombus and uncoagulated blood

In Table A, we have summarized our measurement and unpaired T-test (p-value) results comparing the measured THz absorption constant of thrombus (n=29) and uncoagulated human blood (n=28). The thrombus absorption constant was the one-minute average value (out of 120 continuously measured traces) right after the extracted blood had turned into thrombus. The uncoagulated blood absorption constant was the one-minute average value (out of 120 continuously measured traces) 4 minutes after the extraction of human blood with heparin added. The control group data were taken from a previous study [A1] performed under exactly the same experimental setting and conditions, while the data were re-analyzed to avoid any possible system error.

Table A. Summary of our measurement and unpaired T-test (p-value) results comparing the measured THz absorption constant of thrombus (n=29) and uncoagulated human blood (n=28).

Frequency (THz)	Thrombus absorption constant (cm^{-1})	Standard deviation for thrombus (cm^{-1})	Uncoagulated blood absorption constant (cm^{-1})	Standard deviation for uncoagulated blood (cm^{-1})	p-Value
0.136	91.53512	4.09145	88.31937	3.25977	0.00183
0.200	104.61236	4.75529	110.62954	4.55466	9.62281×10^{-6}
0.27	115.73646	5.54529	120.94855	4.56009	2.92514×10^{-4}
0.34	126.54814	5.73928	129.1893	4.88097	0.06703
0.41	136.82455	6.5007	142.3601	5.10269	7.55523×10^{-4}
0.48	151.80143	6.72593	153.1851	5.48618	0.39936
0.54	161.94568	7.35702	163.93776	5.01947	0.23917
0.61	175.67233	7.72554	177.05358	6.07541	0.45732
0.68	188.47168	7.93866	188.43442	6.85245	0.98496
0.75	203.28865	8.5045	199.93948	5.96335	0.09183
0.82	216.71043	8.98913	213.36989	6.74025	0.11916
0.885	231.46235	8.77756	226.81963	7.66497	0.03818
0.95	241.39882	9.25479	239.68366	8.45499	0.46865
1.02	253.90663	8.36442	247.23516	7.91567	0.00313

T-test results across the whole measured spectrum range (130-1020GHz) comparing early-coagulated blood and uncoagulated blood

In Table B, we have summarized our measurement and unpaired T-test (p-value) results comparing the measured THz absorption constant of early-coagulated (n=29) and uncoagulated (n=28) human blood. The early-coagulated blood absorption constant was the one-minute average value (out of 120 continuously measured traces) with measurement starting at 3 minutes after the extraction of human blood with no heparin added. The uncoagulated blood absorption constant was the one-minute average value (out of 120 continuously measured traces) taking at 4 minutes after the

extraction of human blood with heparin added. The uncoagulated control group data were taken from a previous study [A1] performed under exactly the same experimental setting and conditions, while the data were re-analyzed to avoid any possible system error.

Table B. Summary of our measurement and unpaired T-test (p-value) results comparing the measured THz absorption constant of early-coagulated (n=29) and uncoagulated (n=28) human blood.

Frequency (THz)	Early-coagulated blood absorption constant (cm^{-1})	Standard deviation for early-coagulated blood (cm^{-1})	Uncoagulated blood absorption constant (cm^{-1})	Standard deviation for uncoagulated blood (cm^{-1})	p-Value
0.136	91.92187	2.24409	88.31937	3.25977	9.65959 $\times10^{-6}$
0.20	105.01957	2.71857	110.62954	4.55466	5.45947 $\times10^{-7}$
0.27	116.26109	3.62977	120.94855	4.56009	7.0045 $\times10^{-5}$
0.34	126.94502	3.97573	129.1893	4.88097	0.06183
0.41	137.18878	4.66604	142.3601	5.10269	1.93722 $\times10^{-4}$
0.48	152.18869	4.81613	153.1851	5.48618	0.46885
0.54	162.25435	5.6347	163.93776	5.01947	0.23935
0.61	176.15552	5.72176	177.05358	6.07541	0.56784
0.68	188.96972	6.0687	188.43442	6.85245	0.75584
0.75	203.88854	6.49693	199.93948	5.96335	0.02038
0.82	217.11379	7.20963	213.36989	6.74025	0.04789
0.885	231.94085	6.88999	226.81963	7.66497	0.01036
0.95	241.72282	7.30608	239.68366	8.45499	0.33366
1.02	254.29344	6.60796	247.23516	7.91567	5.67258 $\times10^{-4}$

Blood clotting time versus different systemic hemostatic factors

We analyzed the correlation between the blood clotting time and different systemic

hemostatic factors, including platelet (PLT) count and red blood cell (RBC) count. In this investigation, RBC count was limited within a narrower region in 4 < RBC count <= 5 M/μL while PLT count was limited within the normal region of 100 K/μL to 300 K/μL to avoid the influence of patients with abnormal coagulation function. 18 samples were selected with the two limitations for our correlation analysis. The bivariate Pearson correlation analysis shows no significant correlation between the observed blood clotting time versus the PLT count with a p-value of 0.096. The bivariate Pearson correlation analysis on the other hand shows moderate correlation between the observed blood clotting time versus the RBC count with a p-value of 0.02 and a negative Pearson correlation coefficient (R) of -0.535.

Measurement system stability

The measurement repeatability was tested by injecting bulk water, with the chamber assembled, fixed, water injected, and disassembled three times. The ratio of the standard deviation to the average value was less than 1% in all frequencies between 0.1-1.2THz. The system fluctuation was found to be negligible when compared with the measured difference between samples.

Bivariate Pearson correlation analysis results between the measured absorption constant versus PLT count

We analyzed the correlation between the measured THz absorption constant and platelet (PLT) count. Here the analyzed THz absorption is the one-minute-averaged THz absorption constant measured at 4 minutes right after the extract of blood, when the early stage platelet amplification process completed while blood had not formed the jelly-like thrombus. In this investigation, RBC count was limited within a narrower region in 4 < RBC count <= 5 M/μL while PLT count was limited within the normal region of 100 K/μL to 300 K/μL to avoid the influence of patients with abnormal coagulation function. 18 samples were selected for the early-coagulated blood.

Table C. Summary of the bivariate Pearson correlation analysis results on the measured THz absorption constant of early-coagulated blood versus PLT count.

Frequency (GHz)	p-Value	Correlation Coefficient
136	* 0.03146	0.50777
200	** 0.00153	0.68987

270	** 0.00603	0.62023
340	** 0.00897	0.59654
410	* 0.01556	0.56043
480	** 0.00742	0.6081
540	* 0.01196	0.57816
610	* 0.01756	0.55192
680	* 0.01956	0.54417
750	* 0.01523	0.56187
820	* 0.02135	0.53775
885	* 0.018	0.55017
950	* 0.04129	0.48511
1020	** 0.00798	0.60368

* 0.05>p>0.01; ** 0.01>p>0.001; *** p<0.001

Table D. Summary of the bivariate Pearson correlation analysis results on the measured THz absorption constant of uncoagulated blood versus PLT count. (n=19)

Frequency (GHz)	p-Value	Correlation Coefficient
136	0.40676	-0.20207
200	0.7961	-0.06353
270	0.70543	-0.09283
340	0.79475	-0.06396
410	0.81265	-0.05828
480	0.83594	-0.05094
540	0.68601	-0.09925
610	0.36035	-0.22229

680	0.19132	-0.31343
750	0.75184	-0.07771
820	0.84321	-0.04865
885	0.5972	-0.12951
950	0.32812	-0.23723
1020	0.39371	-0.20762

Table E. Summary of the bivariate Pearson correlation analysis results on the measured THz absorption constant of thrombus versus PLT count. (n=18)

Frequency (GHz)	p-Value	Correlation Coefficient
136	*<br>0.01023	0.5883
200	**<br>0.00553	0.62522
270	**<br>0.00988	0.59046
340	**<br>0.00943	0.59343
410	*<br>0.01867	0.54754
480	**<br>0.00914	0.59535
540	**<br>0.00902	0.59617
610	*<br>0.01559	0.56028
680	*<br>0.01641	0.55671
750	*<br>0.01605	0.55826
820	*<br>0.01701	0.55419
885	*<br>0.01446	0.56545
950	*<br>0.03963	0.48862
1020	*<br>0.01073	0.5852

* 0.05>p>0.01; ** 0.01>p>0.001; *** p<0.001

References

A1. Tseng TF, You B, Gao HC, Wang TD, Sun CK. Pilot clinical study to investigate the human whole blood spectrum characteristics in the sub-THz region. Optics Express 2015;23:9440-9451
